# Bridging Brain Science and Technology: How AI Is Shaping the Future of Neuroimaging in Autism

**DOI:** 10.3390/diagnostics16101425

**Published:** 2026-05-07

**Authors:** Maria-Luiza Băean, Oana Nicu-Canareica, Cristian Constantin Volovăț, Gelu-Adrian Popa, Diana Mihaela Ciuc, Viorel Jinga, Cosmin Medar

**Affiliations:** 1 Department of Fundamental Sciences, Faculty of Midwifery and Nursing, University of Medicine and Pharmacy “Carol Davila”, 050474 Bucharest, Romania; ml_baean@yahoo.com (M.-L.B.); geluadrianpopa@yahoo.com (G.-A.P.); cosmin.medar@umfcd.ro (C.M.); 2 Department of Clinical Laboratory of Radiology and Medical Imaging, CF2 Clinical Hospital, 010024 Bucharest, Romania; 3 Scanexpert Medical Imaging Center, 28 Anastasie Panu Street, 700024 Iași, Romania; 4 Doctoral Program Studies, University of Medicine and Pharmacy “Carol Davila”, 050474 Bucharest, Romania; 5 Department of Radiology, “Grigore T. Popa” University of Medicine and Pharmacy, 16 University Street, 700115 Iași, Romania; 6 Department of Laboratory of Radiology and Medical Imaging, Sf. Ioan Clinical Emergency Hospital, 042122 Bucharest, Romania; 7 Department of Otorhinolaryngology, CF2 Clinical Hospital, 63 Mărăști Boulevard, 011464 Bucharest, Romania; 8 Department of Urology, Clinical Hospital “Prof. Dr. Theodor Burghele”, Faculty of Medicine, University of Medicine and Pharmacy “Carol Davila”, 050474 Bucharest, Romania; viorel.jinga@umfcd.ro; 9 Medical Sciences Section, Academy of Romanian Scientists, 050085 Bucharest, Romania; 10 Department of Clinical Laboratory of Radiology and Medical Imaging, Clinical Hospital “Prof. Dr. Theodor Burghele”, 050664 Bucharest, Romania

**Keywords:** autism spectrum disorder, artificial intelligence, magnetic resonance imaging, corpus callosum, white matter

## Abstract

**Background/Objectives:** Autism Spectrum Disorder (ASD) is associated with structural brain alterations, particularly involving white matter and connectivity. Artificial intelligence (AI) enhances the detection of subtle neuroanatomical changes. This study aimed to characterize structural abnormalities and volumetric patterns in children with ASD using AI-assisted MRI. **Methods:** This retrospective study included 90 children diagnosed with ASD. Brain MRI scans were analyzed using the CE-certified AI platform mdbrain. Structural findings were classified into corpus callosum anomalies, white matter signal abnormalities (WMSA), ventriculomegaly, other abnormalities, or no detectable changes. Group differences were assessed using ANOVA and Kruskal–Wallis tests with Tukey post hoc analysis. Logistic regression, principal component analysis (PCA), and linear discriminant analysis (LDA) were applied. **Results:** WMSA were identified in 23.3% of patients, followed by other anomalies (27.8%), corpus callosum anomalies (8.9%), ventriculomegaly (8.9%), and no abnormalities (31.1%). Total white matter volume was significantly reduced in pathological groups and was the only independent predictor. PCA identified three principal components reflecting shared temporo-parietal covariance, hemispheric asymmetry, and a white matter-related axis. Exploratory LDA demonstrated partial separation among anomaly categories. **Conclusions:** Children with ASD in this cohort showed heterogeneous but partially structured MRI alterations involving both focal and global volumetric changes. Reduced total white matter volume was the most consistent multivariable association with structural abnormalities. AI-assisted morphometric analysis may support structural phenotyping in ASD. These findings are exploratory and require confirmation in larger, prospectively validated cohorts before biomarker applications can be considered.

## 1. Introduction

Autism Spectrum Disorder (ASD) is a complex neurodevelopmental condition characterized by persistent deficits in social communication, restricted interests, and repetitive behaviors. While behavioral assessments remain the gold standard for diagnosis under DSM-5 criteria, recent research increasingly supports the presence of neuroanatomical biomarkers, particularly involving white matter structures and interhemispheric pathways [1].

Among these, the corpus callosum (CC), the largest commissural fiber bundle connecting the cerebral hemispheres, has emerged as a frequently altered structure in individuals with ASD. Structural and diffusion-based MRI studies have consistently reported changes in CC volume, morphology, and microstructural organization, particularly within the genu and splenium, which are key hubs for social cognition and sensorimotor integration [2,3]. These findings align with the broader theory of disrupted neural connectivity in autism, which postulates atypical communication between distributed brain networks [4].

Despite extensive neuroimaging research, no robust structural MRI biomarker has yet entered routine clinical use, as studies over the last two decades have reported diverse findings in ASD, including early brain overgrowth, altered cortical maturation, white matter abnormalities, enlarged extra-axial cerebrospinal fluid spaces, and regional volumetric changes involving temporal, limbic, cerebellar, and callosal structures, many of which remain inconsistent and difficult to replicate across independent cohorts [5,6,7].

A systematic review of 123 structural MRI studies by Pagnozzi et al. found mixed and often contradictory results regarding total brain volume, white matter, corpus callosum morphology, and limbic structures, with strong age-dependent effects and limited reproducibility [5]. More recently, a 25-year voxel-based morphometry meta-analysis by Liloia et al. demonstrated that gray matter volume and gray matter concentration abnormalities in ASD frequently occur in non-overlapping regions, indicating that different analytic methods may yield divergent conclusions [6]. Likewise, a large pediatric review by Halliday et al., covering 172 studies in individuals under 18 years of age, reported substantial between-study heterogeneity in cortical, subcortical, cerebellar, and callosal findings, emphasizing that no single neuroanatomical signature reliably characterizes ASD in childhood [7].

White matter abnormalities represent one of the most frequently discussed but least resolved areas of ASD neuroimaging. Diffusion-based studies have repeatedly implicated the corpus callosum, corona radiata, superior longitudinal fasciculus, and other associative tracts, suggesting altered connectivity and atypical maturation. Nevertheless, the direction of abnormalities has varied considerably across age groups, with some studies reporting increased fractional anisotropy in younger children and others decreased anisotropy in adolescents or adults, implying dynamic developmental trajectories rather than a stable lesion pattern [4]. Similarly, structural studies of the corpus callosum have reported reduced size, segment-specific thinning, hypoplasia, or no significant differences depending on sample composition and methodology [5,7,8]. Findings in temporal and limbic regions, including the hippocampus and parahippocampal cortex, have been equally variable, with reports of both enlargement and reduction across developmental stages [6,7].

At the same time, recent advances in artificial intelligence (AI)-based segmentation and automated volumetry have improved the feasibility of extracting reproducible quantitative biomarkers from routine clinical MRI. Large-scale validation studies have shown that modern deep-learning pipelines can perform robust whole-brain segmentation across heterogeneous scanners and acquisition protocols, including pediatric datasets [9,10].

The integration of artificial intelligence (AI) into neuroimaging has accelerated progress in this domain. AI-powered volumetric tools such as mdbrain (developed by mediaire GmbH) enable rapid, automated segmentation and normative comparison of key brain regions. These platforms reduce inter-rater variability and facilitate the identification of subtle anatomical deviations that may otherwise go unnoticed in routine MRI analysis [6,7].

This study presents findings from a cohort of 90 children with ASD, aged 1 to 16 years, whose MRI scans were analyzed using mdbrain at ScanExpert, a diagnostic imaging center that applies AI-based radiological protocols in pediatric populations. The aim was to investigate the spectrum of structural brain changes in this group, with a particular focus on corpus callosum anomalies and white matter signal abnormalities (WMSA), and to explore whether quantitative regional brain volumes may help refine structural phenotyping and support future biomarker development in ASD.

## 2. Materials and Methods

### 2.1. Participants

This retrospective study included 90 pediatric patients (aged 1 to 16 years, mean age 4.75 ± SD), all previously diagnosed with Autism Spectrum Disorder (ASD) based on DSM-5 clinical criteria. The cohort included 28 females and 62 males, referred for neuroimaging as part of routine diagnostic follow-up, as in Table 1. Patients with confirmed genetic syndromes (e.g., tuberous sclerosis, Rett syndrome) or acquired brain injuries were excluded to limit confounding structural pathology.

### 2.2. MRI Acquisition

All participants underwent brain imaging using a 1.5T or 3T MRI scanner (exact scanner details at ScanExpert center, Siemens Healthineers, Forchheim, Germany). The imaging protocol included:

• High-resolution 3D T1-weighted MPRAGE sequences;
• Axial T2 FLAIR;
• Additional sequences as clinically indicated (e.g., DWI, SWI).

No sedation was used in children who were able to remain still; for younger patients (<5 years), sedation protocols were applied according to pediatric safety standards.

### 2.3. AI-Based Image Analysis

**Figure 1:**
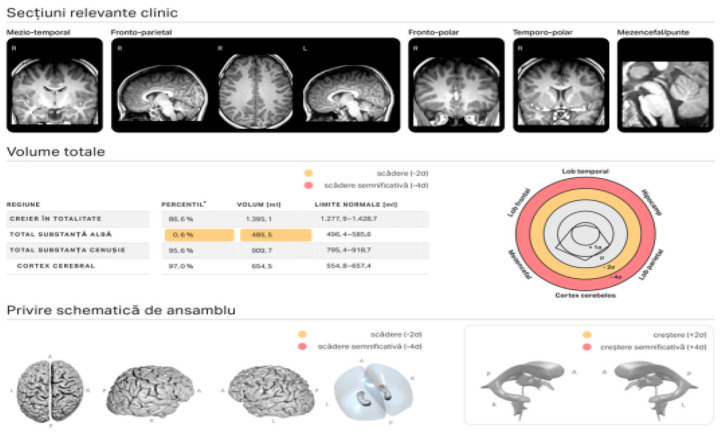
mdbrain volumetry report.

**Figure 2:**
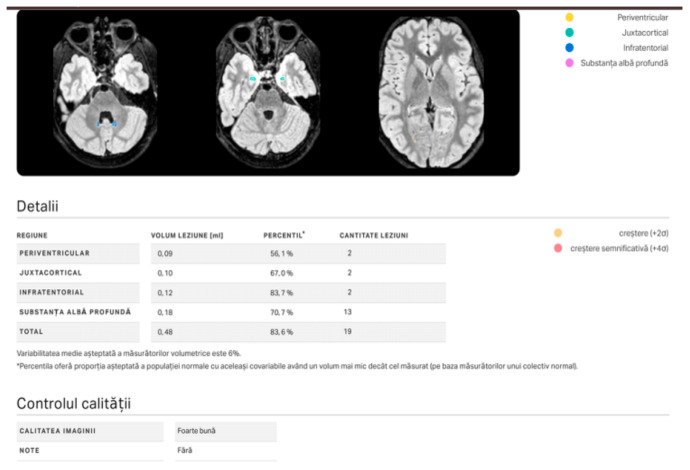
mdbrain lesion report.

Imaging data were post-processed using mdbrain v4.11.0 (mediaire GmbH, Berlin, Germany), a CE-certified AI-based volumetric and lesion-detection platform. The software automatically segmented cortical and subcortical regions, including the corpus callosum, white matter tracts, and periventricular zones (Figure 1 and Figure 2). Normative volumetric comparisons were performed using age- and sex-adjusted reference data embedded in the mdbrain system.

The following structural domains were evaluated:

• Corpus callosum: visual and volumetric assessment for hypoplasia, hypertrophy, or cystic lesions;
• White matter lesions: classified by location as periventricular, deep white matter, juxtacortical, or infratentorial;
• Other anomalies: ventriculomegaly, arachnoid cysts, cisterna magna enlargement, and vascular–neural contacts;

Each scan was reviewed by two neuroradiologists for clinical correlation, with consensus in cases of discrepancy.

### 2.4. Data Categorization and Grouping

Patients were grouped based on the presence of:

• No detectable structural abnormalities (*n* = 28);
• Corpus callosum anomalies (*n* = 8; 6 hypoplastic, 1 cystic, 1 hypertrophic);
• Ventriculomegaly (*n* = 8);
• WMSA (*n* = 21; based on lesion morphology and location);
• Other structural anomalies (*n* = 25; including ponto-cerebellar neurovascular contact, mega cisterna magna, and arachnoid cysts).

Lesions were recorded based on anatomical location as per standardized neuroanatomical atlases. Descriptive statistics were used to summarize frequencies across diagnostic categories.

Statistical analyses were performed using R (version 4.5.3). Continuous variables were expressed as mean ± standard deviation, while categorical variables were presented as frequencies and percentages. Differences between patients without anomalies and those with any structural abnormality were assessed using Student’s *t*-test for parametric data and the Wilcoxon rank-sum test for non-parametric data. Comparisons across the five anomaly groups were performed using one-way analysis of variance (ANOVA) and the Kruskal–Wallis test. When significant differences were identified, post hoc pairwise comparisons were conducted using Tukey’s Honestly Significant Difference (HSD) test.

Normality of distributions within each group was evaluated using the Shapiro–Wilk test. Associations between categorical variables were assessed using chi-square tests with Monte Carlo simulation or Fisher’s exact test when appropriate.

A multivariable binary logistic regression model was performed to evaluate whether regional brain volumetric measurements were independently associated with the presence of major structural MRI abnormalities within the ASD cohort. The dependent variable was anomaly status, coded as no detectable MRI anomaly versus presence of any MRI abnormality. Independent variables entered simultaneously into the model were total white matter volume, right temporal lobe volume, left temporal lobe volume, right precuneus volume, left precuneus volume, right parahippocampal gyrus volume, and left parahippocampal gyrus volume.

The model was estimated using the generalized linear model function with binomial distribution and logit link. Regression coefficients were transformed into odds ratios (ORs) with 95% confidence intervals (95% CIs). Statistical significance of individual predictors was assessed using Wald z statistics.

Model fit was evaluated using Akaike’s Information Criterion (AIC), McFadden’s pseudo-R^2^, Cox–Snell R^2^, and Nagelkerke R^2^. Predicted probabilities were converted into binary classifications using a threshold of 0.50, and model performance was summarized by confusion matrix, overall accuracy, sensitivity, and specificity.

Principal component analysis (PCA) was conducted as an exploratory dimensionality-reduction method to evaluate covariance patterns among the seven volumetric variables.

Linear discriminant analysis (LDA) was additionally performed as an exploratory multiclass classification method to assess whether volumetric MRI measures could discriminate among the five anomaly categories. Internal validation was conducted using leave-one-out cross-validation (LOOCV), and classification accuracy was reported. All statistical tests were two-sided, and *p*-values < 0.05 were considered statistically significant.

## 3. Results

Of the 90 children diagnosed with ASD, 21 patients (23.3%) presented with WMSA, as identified through AI-assisted MRI analysis (Table 2).

**Figure 3:**
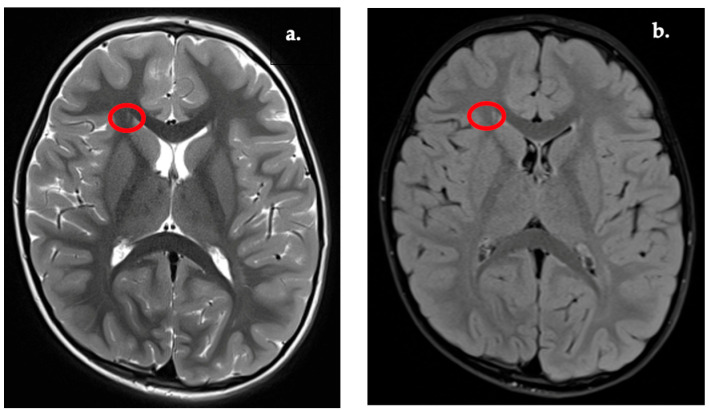
WMSA of the lateral ventricles (frontal horns). (**a**) T2w image. (**b**) FLAIR image.

**Figure 4:**
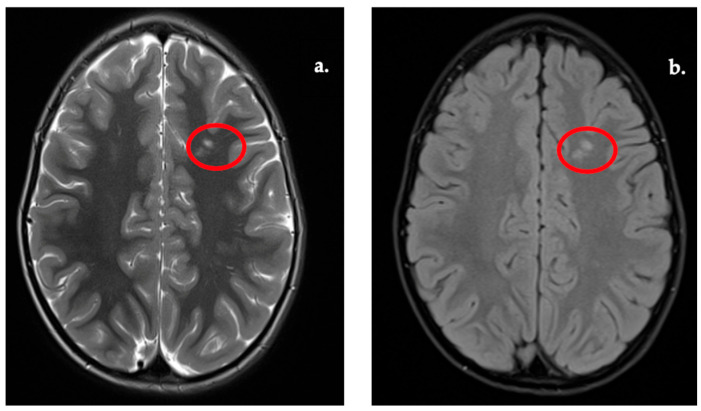
WMSA present in corona radiata. (**a**) T2w image. (**b**) FLAIR image.

**Figure 5:**
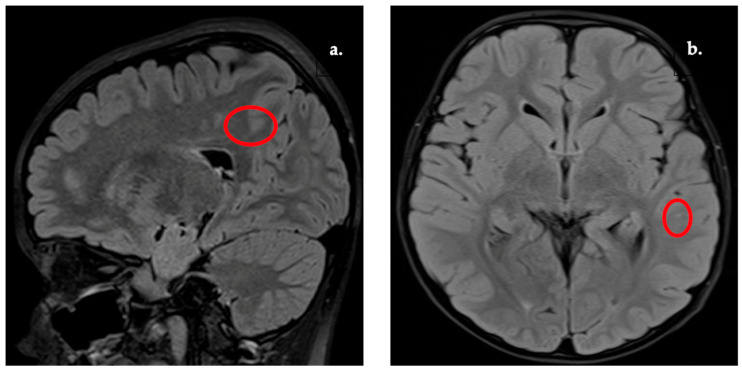
FLAIR images—WMSA in the precuneus (**a**), respectively, in the superior temporal gyri (**b**).

**Figure 6:**
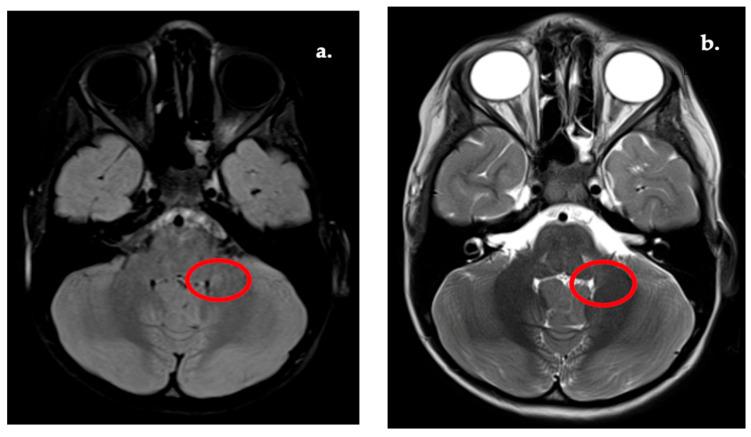
Infratentorial region WMSA—e.g., cerebellum. (**a**) FLAIR image. (**b**) T2w image.

Representative examples of the WMSA subgroup are shown in Figure 3, Figure 4, Figure 5 and Figure 6. These lesion patterns were classified within the WMSA category and subsequently included in volumetric group comparisons. Periventricular and deep white matter lesions were the most frequent patterns (Table 3), whereas juxtacortical and infratentorial lesions were less common. The most common patterns were periventricular (in >50% of WMSA cases), typically involving the atrium and frontal horns of the lateral ventricles (Figure 3). A significant proportion also showed deep white matter lesions, particularly within the centrum semiovale and corona radiata (Figure 4).

Less frequently, juxtacortical involvement was noted—especially in the precuneus, superior temporal gyri, and frontal lobes—regions associated with high-order cognitive processing (Figure 5).

In two children, WMSA extended to infratentorial regions, including the dentate nuclei, splenium of the corpus callosum, and brainstem, raising differential considerations such as adrenoleukodystrophy or NF1-associated FASI (Figure 6).

**Figure 7:**
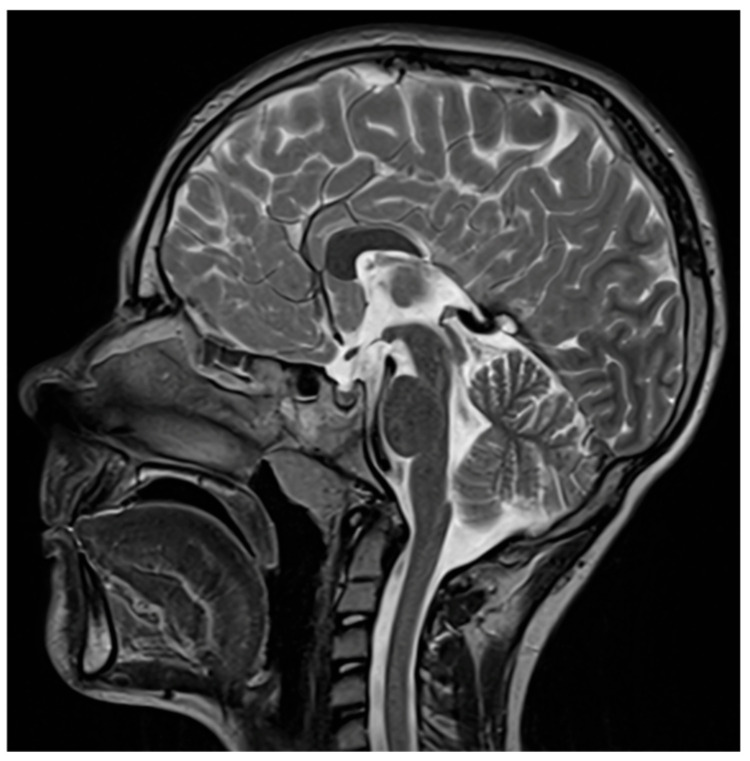
T2w image showing corpus callosum hypoplasia.

**Figure 8:**
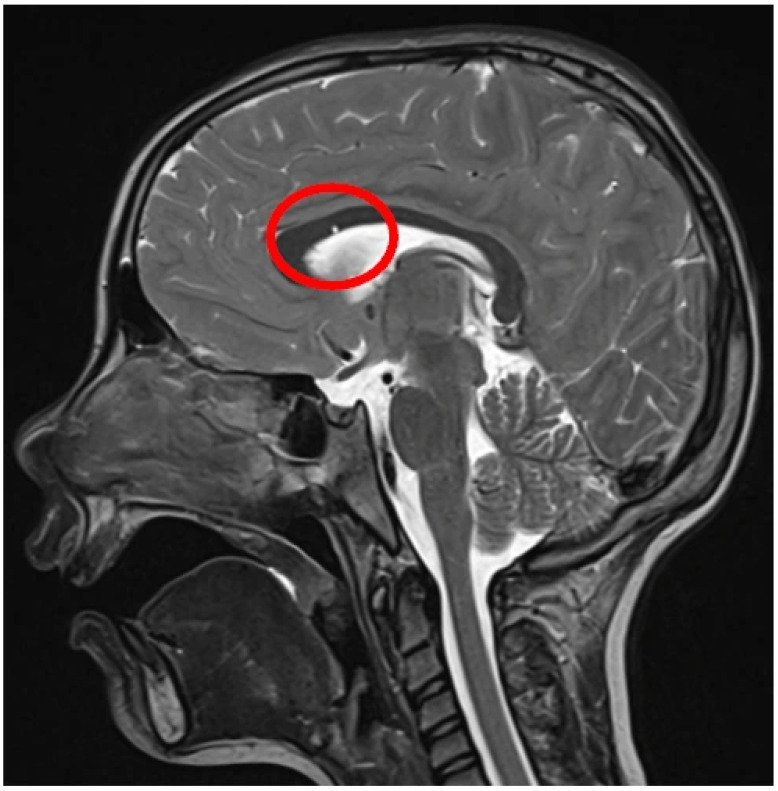
T2w image showing a cyst within the corpus callosum. The red circle indicates the cyst.

**Figure 9:**
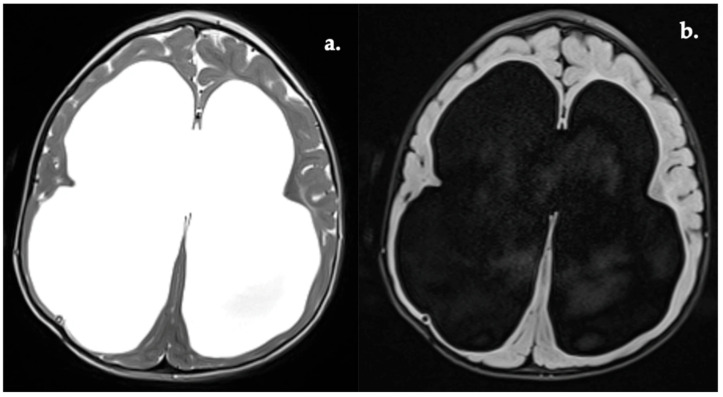
Ventriculomegaly (**a**) T2w image. (**b**) FLAIR image.

A breakdown of lesion localization is presented in Table 3:

In addition to WMSA, the AI analysis revealed:

• No structural brain abnormalities in 28 children (31.1%);
• Corpus callosum anomalies in eight children (8.9%);
  ○ Six hypoplasia (Figure 7), hypertrophy, and one cystic lesion (Figure 8);
• Ventriculomegaly in eight patients (8.9%) (Figure 9).

• Other findings in 25 patients (27.8%), including:
  ○ Twenty-four with neurovascular ponto-cerebellar contact;
  ○ Four with mega cisterna magna;
  ○ Three with arachnoid cysts.

A comparative analysis of structural brain findings according to sex did not demonstrate meaningful differences in distribution across diagnostic categories, suggesting a relatively balanced pattern of abnormalities between male and female patients (Table 4). WMSA, corpus callosum anomalies, ventriculomegaly, and other structural findings were similarly represented across both groups, without statistically significant variation. Likewise, the proportion of patients without detectable abnormalities did not differ substantially by sex.

When stratified by age, no significant difference was observed between patients with abnormal MRI findings and those with normal imaging (Table 5). The mean age was comparable between groups, indicating that the presence of structural abnormalities in this cohort was not age dependent. Overall, these findings suggest that neither sex nor age had a significant impact on the distribution of imaging abnormalities in this ASD population.

When comparing patients without anomalies to those with any structural anomaly, total white matter volume was significantly lower in the anomaly group (*p* < 0.001), representing the most robust global difference (Table 6). Additionally, the left parahippocampal gyrus was significantly reduced in the anomaly group, although the effect size was smaller. All other regions, including both temporal lobes and the precuneus, did not reach statistical significance.

Testing across the five MRI anomaly categories showed statistically significant group differences for all seven volumetric measures using both parametric and non-parametric approaches (Table 7). Total white matter volume differed across groups (*p* < 0.001). Large omnibus differences were also observed for right temporal lobe volume (*p* < 0.001) and for right parahippocampal gyrus volume (*p* < 0.001), with the latter showing the highest ANOVA F*F* statistic among all measures. The precuneus measures also demonstrated robust group differences, including right precuneus (*p* = 0.0015) and left precuneus (*p* < 0.001).

Tukey HSD post hoc tests identified multiple significant pairwise differences, with patterns that were strongly anomaly type dependent (Table 8). For total white matter, WMSA differed from no anomalies by −22.69 (*p* < 0.001), and other anomalies differed from no anomalies by −17.24 (*p* < 0.001).

For right temporal lobe volume, ventriculomegaly differed strongly from no anomalies (−15.29 mL, *p* < 0.001), from WMSA (−16.30 mL, *p* < 0.001), and from corpus callosum anomalies (−19.59 mL, *p* < 0.001), while corpus callosum anomalies exceeded no anomalies (+4.30 mL, *p* < 0.001). For left temporal lobe volume, WMSA exceeded no anomalies (+6.57 mL, *p* = 0.01), and ventriculomegaly was reduced versus no anomalies (−8.91 mL, *p* = 0.01), with further large differences between corpus callosum anomalies and WMSA (−14.58 mL, *p* < 0.001).

For precuneus volumes, ventriculomegaly showed reductions relative to no anomalies (right: −1.33 mL, *p* = 0.009; left: −2.57 mL, *p* < 0.001), while other anomalies showed increases relative to no anomalies (right: +0.80 mL, *p* = 0.03; left: +0.81 mL, *p* = 0.001).

For parahippocampal volumes, corpus callosum anomalies exceeded no anomalies (right: +0.53 mL, *p* < 0.001; left: +0.51 mL, *p* < 0.001), whereas ventriculomegaly was reduced versus no anomalies (right: −0.99 mL, *p* < 0.001; left: −0.29 mL, *p* < 0.001).

Binary logistic regression analysis identified total white matter volume as the only significant predictor of anomaly presence (OR = 0.86, *p* < 0.001); Table 9. Lower white matter volumes were associated with a higher probability of belonging to the pathological group. Thus, our results showed that each 1 mL increase in total white matter volume was associated with an approximately 14% reduction in the odds of belonging to the abnormal MRI group. Variance inflation factor analysis demonstrated low collinearity for total white matter volume (VIF = 1.66) and left temporal lobe volume (VIF = 1.30), but moderate-to-high collinearity for several regional predictors, including right temporal lobe (7.19), right precuneus (8.96), left precuneus (9.45), and right parahippocampal gyrus (9.73).

All other variables, including temporal, precuneus, and parahippocampal volumes, were not statistically significant predictors, indicating that they do not independently discriminate between normal and abnormal cases when considered simultaneously. The model fit was characterized by the following parameters: AIC = 91.0; McFadden’s pseudo-R^2^ = 0.33; Nagelkerke R^2^ = 0.47. Using a 0.50 classification threshold, the model showed an apparent overall accuracy of 84.4%, with sensitivity of 90.3% and specificity of 71.4%. Because performance metrics were derived from the same cohort used to fit the model, these findings should be interpreted as exploratory internal estimates.

PCA was performed as an exploratory dimensionality-reduction method using the seven MRI volumetric variables. The first three principal components explained 82.1% of the total variance (PC1: 48.1%, PC2: 21.0%, PC3: 13.0%) (Table 10).

PC1 was characterized by shared contributions from temporal, precuneus, and parahippocampal regions. PC2 was driven predominantly by left temporal and left parahippocampal measures, whereas PC3 showed the strongest loading for total white matter volume.

**Figure 10:**
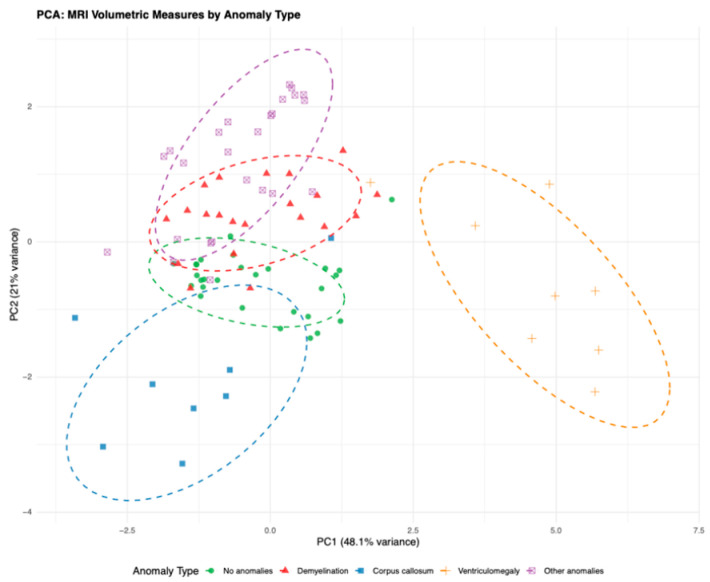
Principal component analysis revealing structural MRI patterns across anomaly types.

The PCA scatter plot (Figure 10) demonstrated partial clustering of anomaly categories, although substantial overlap between groups remained. Separation appeared most evident for ventriculomegaly, while the other categories showed wider dispersion. These findings support exploratory covariance patterns among volumetric measures rather than definitive group discrimination.

**Figure 11:**
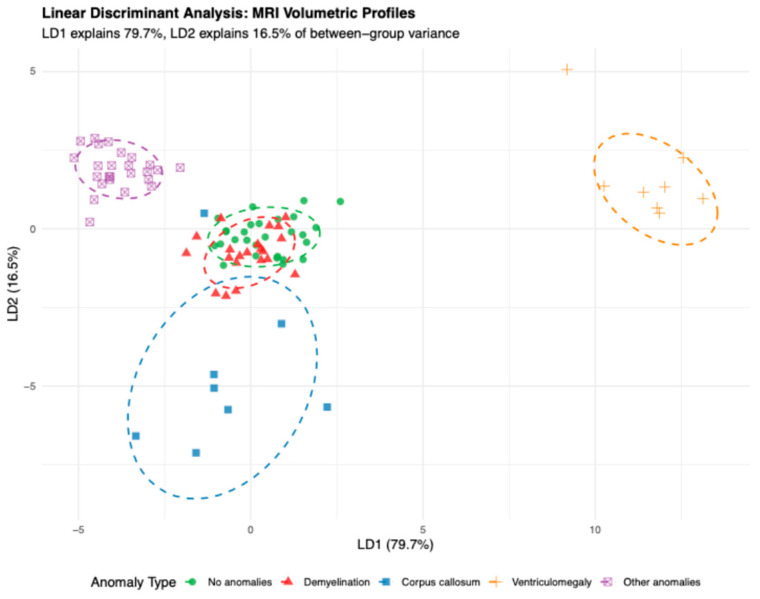
LDA scatter plot.

Linear discriminant analysis (LDA) was performed as an exploratory supervised projection of the five predefined anomaly groups based on seven MRI volumetric predictors (Figure 11). LD1 accounted for 79.7% of the between-group variance, followed by LD2 (16.5%), LD3 (2.7%), and LD4 (1.1%).

Inspection of the discriminant coefficients showed that LD1 was primarily influenced by the right parahippocampal gyrus (−10.742) and left parahippocampal gyrus (+10.575), indicating that relative hemispheric differences in parahippocampal volumes contributed to separation along the first discriminant axis. Smaller contributions were observed for the left precuneus (−0.550) and right precuneus (+0.190). LD2 was also mainly influenced by the left parahippocampal gyrus (−4.855), right parahippocampal gyrus (−1.979), and left precuneus (+0.923), suggesting an additional orthogonal pattern of volumetric variation across groups.

Because the study included a modest total sample size and unequal subgroup sizes, the apparent separation observed on the LDA plot was not interpreted as evidence of a validated classification model. Internal validation using LOOCV achieved an overall classification accuracy of 88.9%, with class-specific accuracies of 92.9% for no anomalies, 76.2% for WMSA, 62.5% for corpus callosum anomalies, 100.0% for ventriculomegaly, and 100.0% for other anomalies. These findings should therefore be regarded as exploratory internally validated estimates requiring confirmation in larger independent cohorts.

## 4. Discussion

The present study evaluated structural MRI findings and automated volumetric measures in a pediatric ASD cohort using an AI-assisted analysis pipeline. The principal findings were threefold.

First, structural abnormalities were common, with white matter signal abnormalities (WMSA) representing the most frequent category (23.3%), followed by other structural findings (27.8%), while corpus callosum anomalies and ventriculomegaly were each identified in 8.9% of patients; 31.1% of children showed no detectable structural abnormalities on MRI.

Second, significant volumetric differences were observed across all seven analyzed brain regions, indicating that structural variability extended beyond focal radiological findings.

Third, reduced total white matter volume emerged as the only independent imaging feature associated with abnormal MRI status in multivariable analysis, whereas exploratory PCA and LDA suggested additional covariance patterns involving temporo-limbic and parahippocampal regions.

These findings are broadly consistent with prior literature indicating that ASD is associated with widespread yet heterogeneous neuroanatomical alterations rather than a single reproducible structural signature [11,12,13].

Previous systematic reviews reported that structural MRI abnormalities in ASD vary substantially across studies, with early brain overgrowth, larger frontal and temporal volumes, increased cortical thickness, and reduced cerebellar or corpus callosum measures described in some pediatric cohorts, but not consistently replicated across age groups [5].

Importantly, the developmental stage appears to be a major source of heterogeneity. Age-stratified studies have shown that some volumetric differences are detectable only within specific developmental windows and may disappear when broad pediatric and adult age ranges are pooled [14]. Lifespan reviews further indicate that age, sex, pubertal status, and hormonal influences contribute to divergent neurodevelopmental trajectories in ASD [15].

Infant studies additionally reported altered CSF volumes, early connectivity differences, and non-linear regional growth patterns in children later diagnosed with ASD [16]. Given the broad age range of the present cohort (1–16 years), part of the observed volumetric variability may therefore reflect developmental timing rather than disorder-specific pathology alone.

In our cohort, these broader patterns were partially reflected in the volumetric analyses, where significant group differences were identified across all seven measured regions. This suggests that ASD-related structural variability may extend beyond focal lesions to involve distributed volumetric differences affecting both global and regional anatomy. At the same time, the heterogeneity of our findings, including WMSA, callosal anomalies, ventriculomegaly, and incidental structural variants, supports the view that ASD likely encompasses multiple neurobiological pathways rather than a unitary anatomical phenotype.

### 4.1. White Matter Abnormalities: WMSA or Atypical Neurodevelopment?

White matter signal abnormalities were the most prevalent imaging finding in our cohort, identified in 23.3% of children, with a predominance of periventricular and deep white matter involvement, particularly within the centrum semiovale and corona radiata. These observations are broadly consistent with prior studies reporting altered white matter organization in ASD, particularly involving the corpus callosum, corona radiata, fornix, and long association tracts [16,17].

However, interpreting such lesions as truly “demyelinating” requires caution. Current evidence suggests that ASD is more accurately characterized by atypical white matter maturation and altered connectivity rather than classical demyelinating pathology [18,19]. Diffusion studies have repeatedly demonstrated reduced fractional anisotropy and altered diffusivity within periventricular and association pathways, supporting disrupted microstructural organization rather than focal myelin loss [20,21].

The lesion distribution observed in our study—dominated by periventricular (38.1%) and deep white matter lesions (28.6%)—closely mirrors regions implicated in long-range connectivity networks. This is compatible with the possibility that these MRI signal changes represent macroscopic correlates of previously described microstructural connectivity abnormalities [22]. At the same time, the relatively high prevalence observed in our cohort, compared with diffusion-based studies, may also reflect increased sensitivity of AI-assisted lesion detection rather than a true increase in pathological burden.

Developmental timing should also be considered. Recent pediatric diffusion studies suggest that white matter alterations in ASD may change direction across age groups, with some younger cohorts demonstrating increased FA values, whereas older children and adolescents more often show reduced values, supporting atypical maturation trajectories rather than a static lesion model [16].

A smaller subset of patients exhibited juxtacortical and infratentorial involvement, including the dentate nuclei and splenium. These patterns are less commonly described in idiopathic ASD and may raise the possibility of overlapping neurodevelopmental or genetic conditions, particularly in the absence of systematic molecular screening [23].

Taken together, our findings support the presence of widespread white matter abnormalities in ASD, while also highlighting uncertainty regarding their biological significance. Rather than representing true inflammatory WMSA, these changes may reflect altered neurodevelopmental trajectories whose detectability is enhanced by automated imaging tools.

### 4.2. Corpus Callosum Alterations: Consistency vs. Variability

Corpus callosum abnormalities were identified in 8.9% of patients in our cohort, predominantly in the form of hypoplasia. This prevalence is lower than that reported in several morphometric studies describing more subtle callosal alterations in ASD populations [24,25].

This discrepancy is likely methodological. Many previous studies relied on group-level volumetry or diffusion metrics capable of detecting microstructural differences not visible on routine structural MRI. In contrast, our analysis focused on morphologically evident abnormalities, which may explain the lower observed prevalence.

A reduced corpus callosum area and thickness, particularly in posterior regions, have been associated with altered total brain volume and white matter organization in adolescents and young adults with ASD [26]. In our study, this relationship is indirectly supported by the reduction in total white matter volume observed in pathological groups, together with the contribution of white matter to PCA component 3, suggesting that global white matter variation may represent a partially independent structural dimension.

Importantly, the literature on corpus callosum alterations in ASD remains inconsistent. While some literature data report reduced volume or thickness, particularly in the genu and splenium [27], other data have failed to identify significant differences or have even described increased dimensions in younger children [28]. This variability likely reflects both methodological heterogeneity and developmental timing.

Accordingly, our findings support the interpretation that callosal abnormalities are present in only a subset of patients and are unlikely to function as standalone biomarkers.

### 4.3. Ventriculomegaly and CSF Spaces: Non-Specific but Recurrent Findings

Ventriculomegaly was observed in 8.9% of patients, without significant association with age or sex. This finding is broadly consistent with previous reports describing increased ventricular or extra-axial CSF volumes in subsets of children with ASD [19,29].

However, the relatively modest prevalence in our cohort suggests that ventriculomegaly is unlikely to represent a core feature of ASD. It may instead reflect secondary neurodevelopmental processes, including altered cortical growth dynamics or CSF circulation patterns [30].

This interpretation is partially consistent with our post hoc analyses, in which the ventriculomegaly subgroup demonstrated some of the largest reductions in temporal lobe and precuneus volumes. Nevertheless, ventricular enlargement is a highly non-specific finding and is also observed in neurotypical pediatric populations [31].

Population imaging studies reported incidental findings in approximately 16–21% of children undergoing MRI, with only a small minority requiring follow-up or urgent referral [32,33]. Therefore, mild ventriculomegaly should be interpreted cautiously and may represent background anatomical variability rather than an ASD-specific marker.

### 4.4. Incidental Structural Findings: Expanding the Spectrum of Neurodevelopmental Variability

A substantial proportion of patients in our cohort (27.8%) exhibited additional structural findings, including neurovascular ponto-cerebellar contacts, mega cisterna magna, and arachnoid cysts. These findings are often considered incidental and are frequently excluded from ASD-focused analyses [34].

However, pediatric MRI studies indicate that incidental abnormalities are common in the general population. Meta-analytic data reported intracranial cysts in approximately 10.2% of healthy children, nonspecific white matter hyperintensities in 1.9%, and Chiari I malformation in 0.8%, while only 2.6% required follow-up, and clinically actionable lesions were rare (~0.4%) [32]. Other cohorts similarly reported incidental findings in more than one-fifth of children [33]. Pediatric arachnoid cyst prevalence has been estimated at approximately 2.6%, with most cases asymptomatic [35].

These background prevalence rates suggest that at least a proportion of the ancillary findings observed in our ASD cohort may represent coincidental anatomical variants rather than direct manifestations of ASD biology.

At present, the absence of a typically developing control group prevents definitive conclusions regarding overrepresentation in ASD. Their interpretation should therefore remain exploratory pending controlled and longitudinal investigation.

### 4.5. AI-Assisted Neuroimaging: Increased Sensitivity, Persistent Uncertainty

The use of AI-based analysis through mdbrain enabled systematic detection and classification of structural abnormalities, contributing to the relatively high rate of findings in our cohort, including subtle white matter signal changes that may be overlooked during conventional visual assessment.

This observation aligns with broader neuroimaging trends in which machine learning tools may increase sensitivity to subtle structural variation [36]. However, increased sensitivity does not necessarily translate into improved clinical specificity.

Automated morphometry in children presents specific technical challenges, including smaller structures, motion artifacts, rapidly changing brain geometry, and age-dependent tissue contrast [37]. Pediatric normative volumetry studies have shown promising performance, but often rely on single-scanner datasets and strict quality control, with uncertain generalizability to heterogeneous clinical settings [38]. Likewise, validation studies indicate that while AI-based ventricular quantification may outperform manual segmentation, external validation across scanners and age groups remains essential [39].

Scanner-related variability is an additional consideration. Multi-site ASD datasets have shown that field strength, acquisition parameters, and site effects may partially mask or mimic biological group differences. Harmonization approaches such as ComBat improved classification performance in ABIDE-derived datasets [40], although inappropriate adjustment strategies may also remove biologically relevant variance if age and sex are not jointly modeled [41]. Independent comparisons across 1.5T and 3T systems found ComBat to reduce scanner-related bias more effectively than intensity-normalization methods alone [42].

Because MRI examinations in our study were obtained on both 1.5T and 3T systems under routine clinical conditions, residual scanner-related technical variance cannot be excluded. Accordingly, our findings illustrate both the strengths and limitations of AI-assisted neuroimaging. While such tools may enhance detection and standardization, they may also amplify clinically ambiguous findings. AI should therefore be integrated as a complementary tool within multidisciplinary assessment rather than as a standalone diagnostic solution.

### 4.6. Study Limitations

Several limitations of this study include the single-center retrospective design, which may limit generalizability, and the modest sample size with unequal subgroup distribution, which may reduce statistical power and increase model instability. The absence of an independent external validation cohort means that the multivariate findings, particularly those derived from logistic regression and LDA, should be interpreted as internally generated exploratory results requiring confirmation in larger prospective datasets. The cross-sectional design precludes causal or developmental inferences. The absence of a typically developing control group limits interpretation to within-cohort ASD comparisons. In addition, AI-assisted volumetric measurements may be influenced by image quality, motion artifacts, and software-specific processing assumptions. MRI examinations were obtained in routine clinical practice on different scanner platforms, but detailed scanner-specific acquisition metadata were not consistently available for retrospective harmonization analyses. Therefore, scanner-related technical variability may have influenced volumetric estimates. Finally, detailed clinical and longitudinal outcome data were not consistently available for imaging–phenotype correlation analyses.

### 4.7. Clinical Relevance

The clinical relevance of the present findings lies in demonstrating that structural MRI abnormalities and reproducible volumetric differences are common within a pediatric ASD cohort and can be quantitatively characterized using an AI-assisted workflow. In particular, reduced total white matter volume emerged as an imaging feature associated with abnormal MRI findings. Although not sufficient for standalone diagnosis or stratification, these results suggest that quantitative MRI markers may support risk profiling, selection of patients for further evaluation, and future multimodal ASD subgrouping when combined with detailed clinical data.

## 5. Conclusions

In this pediatric ASD cohort, AI-assisted brain MRI analysis identified a heterogeneous spectrum of structural findings, with white matter signal abnormalities representing the most frequent category, followed by incidental structural variants, corpus callosum anomalies, and ventriculomegaly. Significant volumetric differences were observed across all analyzed brain regions, indicating that structural variability extended beyond focal radiological findings. Reduced total white matter volume was the only independent imaging feature associated with the presence of abnormal MRI findings in multivariable analysis. Exploratory multivariate analyses further suggested covariance patterns involving temporo-limbic and parahippocampal regions.

Collectively, these results indicate that structural brain alterations are common but heterogeneous in children with ASD and can be quantitatively characterized using AI-assisted MRI workflows. Although these findings are not sufficient for standalone diagnostic use, they support the potential role of automated morphometric assessment as an adjunctive tool for structural phenotyping and future multimodal stratification studies in ASD.

Future directions should include longitudinal studies to evaluate the progression of these abnormalities, integration with cognitive-behavioral phenotypes, and comparative analysis with neurotypical controls. As imaging algorithms evolve, AI could serve as a central node in a multimodal diagnostic ecosystem, combining structural, functional, and molecular data to redefine how we understand and manage autism spectrum conditions.

## Figures and Tables

**Table 1:** Baseline demographic characteristics of the study population.

Variable	Total (*n* = 90)
Age (years), mean ± SD	4.75 ± SD
Age range (years)	1–16
Male, *n* (%)	62 (68.9%)
Female, *n* (%)	28 (31.1%)

**Table 2:** Distribution of structural brain findings in ASD cohort.

Imaging Finding	*n* (%)
No detectable abnormalities	28 (31.1%)
WMSA	21 (23.3%)
Corpus callosum anomalies	8 (8.9%)
Ventriculomegaly	8 (8.9%)
Other structural abnormalities	25 (27.8%)

**Table 3:** Distribution of WMSA by anatomical location.

WMSA	*n* (%)	Representative Regions
Periventricular	8 (38.1%)	Anterior horn, atrium of lateral ventricles
Deep white matter	6 (28.6%)	Centrum semiovale, corona radiata
Juxtacortical	4 (19.0%)	Precuneus, superior frontal and temporal gyri
Infratentorial	2 (9.5%)	Dentate nuclei, splenium, brainstem
Extensive/mixed	1 (4.8%)	Multiple regions

**Table 4:** Comparison of structural findings by sex.

Variable	Male (*n* = 62) *n* (%)	Female (*n* = 28) *n* (%)	*p*-Value
WMSA	15 (24.2)	6 (21.4)	0.78
Corpus callosum anomalies	6 (9.7)	2 (7.1)	0.69
Ventriculomegaly	6 (9.7)	2 (7.1)	0.69
Other abnormalities	17 (27.4)	8 (28.6)	0.91
No abnormalities	18 (29.0)	10 (35.7)	0.52

**Table 5:** Comparison of age between patients with and without structural abnormalities.

Variable	Abnormal MRI (*n* = 62) Mean ± SD	Normal MRI (*n* = 28) Mean ± SD	Mean Difference (0–1)	*p*-Value
Age (years)	4.9 ± 4.08	4.4 ± 3.92	0.5	0.63

Table legend: SD—standard deviation. Statistical analysis: Categorical variables were compared using the chi-square test; continuous variables using Student’s *t*-test.

**Table 6:** Descriptive statistics: No anomaly vs. With anomaly.

Variable	Mean (No Anomaly)	Mean (With Anomaly)	*p* (*t*-Test)	*p* (Wilcoxon)
Total White Matter	501.46	483.26	<0.001	<0.001
Right Temporal Lobe	89.79	89.31	0.630	0.392
Left Temporal Lobe	82.63	84.48	0.258	0.124
Right Precuneus	14.73	14.89	0.431	0.076
Left Precuneus	14.75	14.71	0.821	0.337
Right Parahippocampal Gyrus	3.41	3.35	0.324	0.845
Left Parahippocampal Gyrus	3.42	3.32	0.048	0.007

**Table 7:** ANOVA and Kruskal–Wallis (five groups).

Variable	ANOVA F	*p*-Value	Kruskal χ^2^	*p*-Value
Total White Matter	9.04	<0.001	29.89	<0.001
Right Temporal Lobe	64.71	<0.001	30.50	<0.001
Left Temporal Lobe	12.91	<0.001	33.08	<0.001
Right Precuneus	7.61	<0.001	17.50	0.0015
Left Precuneus	32.81	<0.001	32.38	<0.001
Right Parahippocampal Gyrus	90.99	<0.001	32.42	<0.001
Left Parahippocampal Gyrus	40.73	<0.001	52.19	<0.001

**Table 8:** Significant Pairwise Differences (Tukey HSD Post hoc Tests).

Variable	Comparison	Mean Difference (mL)	95% CI	*p*-Value
Total White Matter	WMSA vs. No anomalies	−22.69	[−34.00, −11.38]	<0.001
	Other anomalies vs. No anomalies	−17.24	[−28.02, −6.45]	<0.001
Right Temporal Lobe	Ventriculomegaly vs. No anomalies	−15.29	[−18.46, −12.12]	<0.001
	Ventriculomegaly vs. WMSA	−16.30	[−19.59, −13.02]	<0.001
	Ventriculomegaly vs. Corpus callosum	−19.59	[−23.54, −15.63]	<0.001
	Corpus callosum vs. No anomalies	+4.30	[1.13, 7.47]	0.003
Left Temporal Lobe	WMSA vs. No anomalies	+6.57	[1.08, 12.07]	0.011
	Ventriculomegaly vs. No anomalies	−8.91	[−16.54, −1.28]	0.014
	Corpus callosum vs. WMSA	−14.58	[−22.49, −6.67]	<0.001
Right Precuneus	Ventriculomegaly vs. No anomalies	−1.33	[−2.43, −0.23]	0.009
	Other anomalies vs. No anomalies	+0.80	[0.05, 1.56]	0.031
Left Precuneus	Ventriculomegaly vs. No anomalies	−2.57	[−3.38, −1.76]	<0.001
	Other anomalies vs. No anomalies	+0.81	[0.25, 1.36]	0.001
Right Parahippocampal Gyrus	Corpus callosum vs. No anomalies	+0.53	[0.34, 0.71]	<0.001
	Ventriculomegaly vs. No anomalies	−0.99	[−1.18, −0.81]	<0.001
Left Parahippocampal Gyrus	Corpus callosum vs. No anomalies	+0.51	[0.31, 0.70]	<0.001
	Ventriculomegaly vs. No anomalies	−0.29	[−0.49, −0.10]	<0.001

**Table 9:** Binary logistic regression.

Variable	OR	*p*-Value
Total White Matter	0.863	<0.001
Right Temporal	0.857	0.26
Left Temporal	0.996	0.91
Precuneus Right	1.85	0.37
Precuneus Left	2.40	0.26
Parahippocampal Right	0.10	0.37
Parahippocampal Left	28.76	0.15

**Table 10:** Principal Component Loadings for MRI Volumetric Measures.

Variable	PC1	PC2	PC3
Total White Matter (mL)	−0.158	−0.439	0.735
Right Temporal Lobe (mL)	−0.499	0.060	−0.217
Left Temporal Lobe (mL)	−0.200	0.564	−0.111
Right Precuneus (mL)	−0.375	0.249	0.391
Left Precuneus (mL)	−0.478	0.200	0.220
Right Parahippocampal Gyrus (mL)	−0.487	−0.199	−0.293
Left Parahippocampal Gyrus (mL)	−0.282	−0.587	−0.337

## Data Availability

The original contributions presented in this study are included in the article. Further inquiries can be directed to the corresponding authors.
